# Community-Acquired *Candida albicans* Empyema Leading to Tension Physiology: A Case Report

**DOI:** 10.5811/cpcem.19426

**Published:** 2024-08-01

**Authors:** Jason Cinti, Paula Gomez, Suneil Agrawal

**Affiliations:** Desert Regional Medical Center, Department of Emergency Medicine, Palm Springs, California

**Keywords:** *case report*, *empyema thoracis*, *tension empyema*, *Candida albicans*, *severe hyponatremia*

## Abstract

**Introduction:**

A tension empyema, in which purulent material accumulates in the chest cavity and leads to cardiopulmonary dysfunction, is a rare complication of empyemas. Moreover, fungal empyemas that grow *Candida albicans* and cause tension physiology have not yet been previously described.

**Case Report:**

In this report, we present an immunocompetent 30-year-old male who presented to the emergency department with worsening shortness of breath and was found to have a left-sided fungal empyema causing tension physiology. Left chest thoracostomy yielded approximately 4 liters of purulent fluid. Pleural cultures eventually grew *C albicans*, and after antifungal therapy, surgical decortication of the lung, and a prolonged intensive care unit stay, the patient was discharged home in stable condition.

**Conclusion:**

While mortality from *C albicans* empyemas that cause respiratory compromise is exceedingly high, our case highlights that aggressive management with rapid chest thoracostomy and antifungal therapy can lead to a favorable outcome.

Population Health Research Capsule What do we already know about this clinical entity?

*Tension empyemas are large accumulations of purulent material in the chest cavity that entrap the lung, leading to cardiopulmonary dysfunction.*

What makes this presentation of disease reportable?

*Fungal empyemas that grow* Candida albicans *and cause tension physiology have not been previously described.*

What is the major learning point?

*Tension physiology and cardiovascular collapse are rare but possible complications of empyemas; thus, it is critical that physicians quickly identify and treat it.*

How might this improve emergency medicine practice?

*Aggressive management with chest thoracostomy and antifungal therapy can lead to a favorable outcome despite the high mortality associated with this disease process.*

## INTRODUCTION

Tension empyema describes a large accumulation of purulent material in the chest cavity, which then entraps the lung and compresses mediastinal structures. This causes cardiopulmonary dysfunction by preventing adequate ventilation and can lead to severely reduced cardiac output via increased intrathoracic pressures and decreased venous return, resulting in life-threatening cardiac arrest.^1^

Empyemas are often polymicrobial, composed of both aerobic and anaerobic bacteria, and are most commonly caused by pneumonia and parapneumonic effusion.^2^ ^,^ ^3^ Those that grow fungi are particularly rare. *Candida albicans* empyema has previously been described as a potential complication of community-acquired pneumonia.^4^ However, most candidal empyemas have been diagnosed in patients with preceding abdominal and intrathoracic surgeries or patients with esophageal rupture.^5^ To our knowledge, there have been no cases in the literature that describe candida empyema that demonstrate tension physiology. In this case we present an immunocompetent patient with no reported past medical history who presented to the emergency department (ED) with a chief complaint of shortness of breath. He was found to have a tension empyema that grew *C albicans*.

## CASE REPORT

A 30-year-old male with no reported past medical history presented to the ED by ambulance with the chief complaint of shortness of breath. He reported progressively worsening dyspnea and a productive cough for the prior 6–8 weeks. He was given an albuterol breathing treatment by emergency medical services (EMS) with no relief of his symptoms, and he was placed on a non-rebreather mask at 15 liters per minute (L/min) by EMS due to an initial oxygen saturation of 80%. The patient denied recent fevers, chills, chest pain, nausea, vomiting, abdominal pain, urinary complaints, leg swelling, recent sick contacts, or recent travel. He denied a history of intravenous drug use; however, he did endorse daily tobacco smoking and heavy alcohol use, particularly beer. His mother provided additional history, stating that the patient had been drinking over 10 beers per day for the prior several weeks with minimal food intake.

Initial vitals in the ED were notable for tachycardia at 115 beats per minute, tachypnea at 30 breaths per minute, and hypoxia at 82% on a 15 L/min non-rebreather mask. He was afebrile, and his blood pressure was 135/85 millimeters of mercury (mm Hg). Physical exam revealed a toxic-appearing, uncomfortable male in moderate respiratory distress. He was diaphoretic. He had decreased breath sounds over the left lower lobe and clear breath sounds on the right. His chest wall was asymmetrical, with the left chest appearing larger and more protruding than the right chest. Laboratory blood work taken on arrival revealed a leukocytosis of 20 × 10^3^ white blood cells per microliter (10^9^/L) (reference range 4.2–10.8 × 10^9^/L), severe hyponatremia at 104 milliequivalents (mEq)/L (137–145 mEq/L), a lactic acidosis at 3.22 millimoles (mmol)/L (mmol/L) (0.8–2.0 mmol/L), and a negative blood alcohol level. The rest of the basic metabolic panel revealed a potassium of 5.3 mEq/L (3.4–5.0 mEq/L), chloride of 69 mEq/L (98–107 mEq/L), bicarbonate of 21 mEq/L (21–30 mEq/L), glucose 138 mg/dL (70–110 mg/dL), calcium of 7.0 milligrams per deciliter (mg/dL (7.8–9.8 mg/dL)), blood urea nitrogen of 8.0 mg/dL (9.0–20 mg/dL), creatinine of 0.3 mg/dl (0.7–1.3 mg/dL), and an albumin of 2.7 grams (g)/dL (3.5–5.0 g/dL). The urine drug screen was positive only for tetrahydrocannabinol.

**Image 1.**
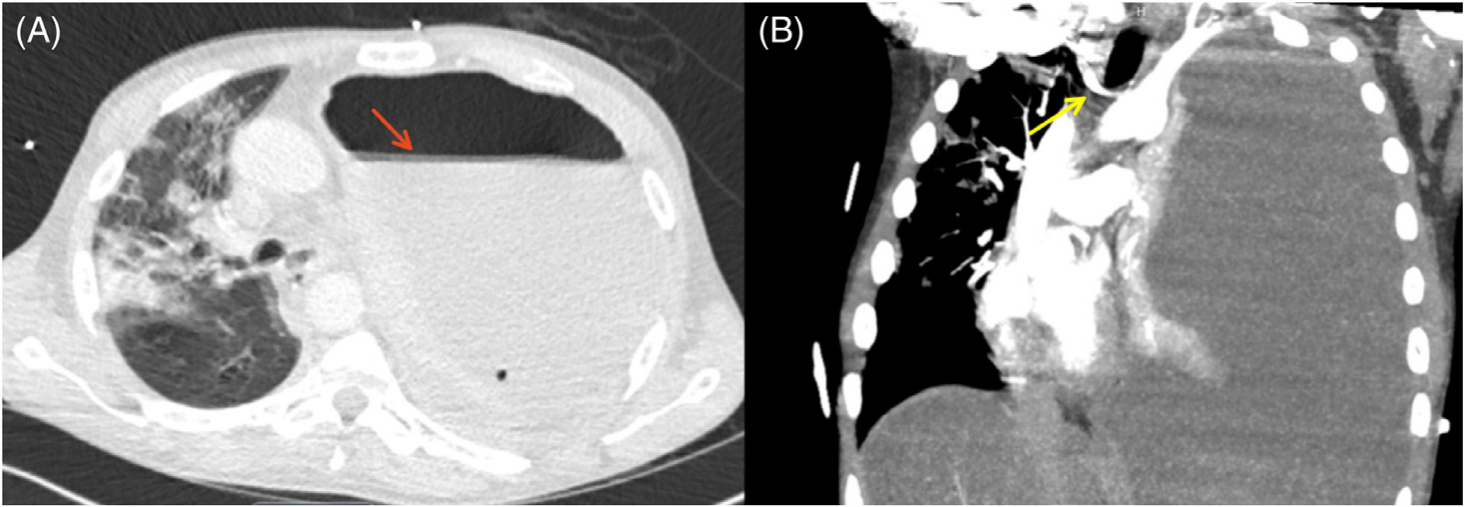
A. Axial view of initial computed tomography chest showing large left hydropneumothorax with complete left lung collapse (red arrow). B. Coronal view of significant rightward shift of the trachea and mediastinum (yellow arrow).

The patient was started on broad spectrum antibiotics with vancomycin and cefepime in the ED. Due to delays in radiographs, the patient had a computed tomography (CT) of the chest with intravenous contrast to further investigate the cause of his difficulty breathing. The CT was immediately reviewed by the emergency physician, and the patient was found to have a large collection of fluid and gas in the left chest, with a deviated trachea and extensive rightward deviation of the heart (Image 1). While not true tension physiology given the patient’s indolent onset of symptoms (6–8 weeks) and initial hemodynamic stability, as tension physiology typically describes a more acute condition involving hemodynamic collapse, such findings on CT were certainly concerning for impending tension physiology.

Based on the findings on CT and his increasing work of breathing, he was intubated emergently. His oxygen saturation prior to intubation was 84% on a 15 L/min non-rebreather mask. He became hypotensive post-intubation after sedation and was started on norepinephrine infusion. Given the large amount of fluid and air seen on CT, it was initially thought that the patient may have had a diaphragmatic hernia with extrusion of the stomach into the left thorax. For this reason, general surgery was consulted. A subsequent CT of the abdomen and pelvis showed no diaphragmatic rupture or abdominal involvement. The surgery resident performed a left-sided thoracostomy, with immediate output of four liters of thick, purulent, and highly malodorous material. Hemodynamics improved after chest tube insertion, with improvement in his blood pressure from 88/55 mm Hg to 113/64 mm Hg. The patient was admitted to the intensivist care unit (ICU) on mechanical ventilation and remained on 15 micrograms/min of norepinephrine.

**Image 2.**
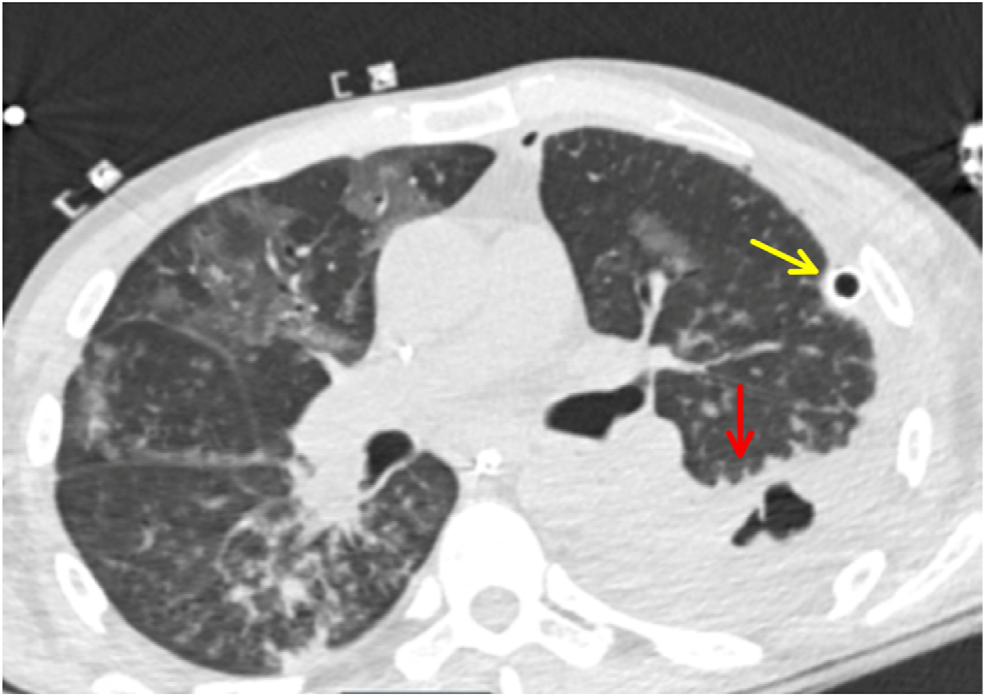
Repeat axial computed tomography chest with contrast on day six of intensive care unit admission showing persistent large empyema reduced in size (red arrow) with resolution of mediastinal shift. Chest tube still in place (yellow arrow), and bilateral ground-glass opacities present.

During his inpatient stay, pleural fluid anaerobic cultures grew *Prevotella oris*, suggesting oral microbes and aspiration pneumonia with parapneumonic effusion and empyema. Pleural fluid and sputum cultures as well as blood cultures also grew *C albicans*. On repeat CT chest one day later, the patient had a persistent empyema. To evacuate the empyema, cardiothoracic surgery was consulted and performed a left thoracoscopy with total decortication, lysis of adhesions, and total parietal pleurectomy. Postoperative CT chest showed persistent but reduced empyema and improved mediastinal shift (Image 2).

The patient was successfully extubated six days after the cardiothoracic procedure, the chest tubes were removed, and he was downgraded from the ICU to the telemetry unit 15 days after admission. His hyponatremia resolved on day nine after admission with careful fluid restriction and maintenance fluids. Owing to rapid initial overcorrection, desmopressin and dextrose 5% in water were used to prevent central pontine myelinolysis.

Repeat CT chest prior to discharge showed residual airspace densities in the lungs with improved aeration compared to the prior study, persistent atelectasis at the left lower lobe with interval re-expansion of the left upper lobe, and diffuse pleural thickening in the left hemithorax with loculated left hydropneumothorax, significantly smaller compared to the prior study. He was discharged home with a walker 24 days after admission with follow-up with cardiothoracic surgery in two weeks.

## DISCUSSION

In this report we describe a case of *C albicans* empyema causing impending tension physiology in a 30-year-old male with a history of alcoholism, with over 4 liters of purulent, malodorous output after initial tube thoracostomy. There are only a few case reports in the literature that describe tension empyemas, and none that we are aware of that describe tension physiology from a fungal empyema. Ooko et al discuss a tension empyema causing cardiac arrest in a young, immunocompromised patient with HIV and tuberculosis, with ∼2900 milliliters (mL) of pus output after thoracostomy.^6^ Ahern et al describe a 28-year-old female with HIV with post-intubation cardiac arrest and output of ∼1500–2000 mL of purulent exudate.^7^ Bramley et al discuss a 42-year-old male with diabetes mellitus type 1 and trisomy 21 who presented in pulseless electrical activity and a hypo-inflated left hemithorax with absent breath sounds, with ∼900 mL of malodorous pus output.^8^ None of these cases were of fungal etiology. Until our case, the largest fungal empyema described in the literature was by Srinivasnakshatri et al in 2014, with ∼2500 mL of fluid that grew *C krusei* and *C tropicalis* drained over 48 hours.^9^ The immediate drainage of 4 liters of pus growing *C albicans* in our case is thus unprecedented.

The case is also unusual in that empyemas that grow *C albicans* are often a result of esophageal perforation, fistula formation, or preceding abdominal and intrathoracic surgeries.^4^ In the first retrospective analysis of 67 patients with fungal empyema in Taiwan by Ko et al, the most common underlying cause (30%) was abdominal disease, especially prior abdominal surgery or gastrointestinal perforation.^5^ Our patient did not have a preceding surgery or gastrointestinal perforation. It is likely that he had an aspiration event owing to his excessive alcohol use that ultimately led to pneumonia with subsequent parapneumonic effusion, which is further reflected by the fact that the pleural fluid also grew *Prevotella*, predominantly an oral microbe. This is a common mechanism: in one retrospective study of 63 patients with *Candida* empyema thoracis, 55.6% of these were from contiguous infection.^10^

In another retrospective analysis of 81 patients, *Candida* empyema was from an intrathoracic source in 51% of patients.^4^ The second most common underlying cause of fungal empyema in the analysis by Ko et al was bronchopulmonary infections at 22%.^5^ However, given that our patient was found to have candidemia, it is also possible that an existing trigger in the pleural space, such as a hemothorax, chylothorax, or hydrothorax, became infected due a systemic infection via hematogenous spread. This is less commonly reported in the literature, with 2% of patients having concurrent candidemia in Senger et al and 27% having concurrent fungemia in Ko et al.^4^ ^,^ ^5^

Furthermore, mortality from *C albicans* empyemas is extremely high and is more likely to occur in older patients and those in an immunocompromised state. In the retrospective analysis previously discussed by Ko et al, mortality in patients with fungal empyema was 73%.^5^ Lin et al’s retrospective study of patients with *Candida* empyema thoracis found mortality to be 61.9%.^10^ *C albicans* empyema was most common. Patients who presented with respiratory failure, as our patient did, were associated with higher mortality. In conjunction, these two studies show that fungal empyema of any kind is associated with very high mortality.

An immunocompromised state appears to play a role in *Candida* empyemas as well. We saw this association previously in patients with tension empyemas without fungal etiologies who were immunocompromised secondary to HIV, tuberculosis, and type 1 diabetes mellitus.^6^ ^,^ ^8^ Two other case reports describe patients with *C albicans* empyemas who were immunocompromised secondary to carcinoma, specifically, esophageal carcinoma, and one of which did not involve gastrointestinal perforation.^11^ ^,^ ^12^ Fortunately, our young patient with no significant medical history other than alcohol use survived the tension empyema and was eventually discharged from the hospital. In our case, however, it is likely that the patient developed an immunocompromised state not from HIV but from chronic alcoholism and malnutrition.

## CONCLUSION

Tension physiology and cardiovascular collapse are rare complications of empyema thoracis. This case report is unique in that this was a community-acquired *C albicans* infection, not associated with gastrointestinal perforation, HIV, or recent surgery, and demonstrated impending tension physiology on physical exam and imaging. Due to the high risk of mortality associated with pulmonary fungal infections, it is critical that physicians quickly identify tension physiology and keep a broad differential when choosing appropriate antibiotic treatment.
